# Deep Learning Discriminates Seizures from Normal Brain Oscillations in the Electroencephalogram of a Rat Model of Post-traumatic Epilepsy

**DOI:** 10.1523/ENEURO.0032-26.2026

**Published:** 2026-05-07

**Authors:** Sean Tatum, Jeremy A. Taylor, Katie Waldon, Anthony J. Garcia, Aaron Witt, Zachariah Z. Smith, Slavka Ryger, Andrew Zayachkivsky, F. Edward Dudek, Daniel S. Barth

**Affiliations:** ^1^Department of Psychology and Neuroscience, University of Colorado, Boulder, Colorado 80309; ^2^Department of Neurosurgery, University of Utah School of Medicine, Salt Lake City, Utah 84108

**Keywords:** CNN, SWD

## Abstract

This study used machine learning to objectively identify seizures in the electroencephalogram of a model of post-traumatic epilepsy based on fluid percussion injury in male rats. We applied transfer learning to a neural-network trained and tested on three potentially distinct electroencephalographic phenotypes: (1) late-onset convulsive seizures associated with rare post-traumatic epilepsy, (2) early-onset convulsive seizures that often occurred after sham or injury treatment (independent of post-traumatic epilepsy), and (3) spike-wave discharges (SWDs), which occurred in both injured and sham-control rats. The neural network was able to detect seizure events within individual animals and across different cohorts and showed that early and late seizures have similar electroencephalographic phenotypes. Additionally, cross-over training and testing on SWDs from injured and sham-control rats distinguished a convulsive seizure phenotype from normal SWDs. Convolutional neural network modeling of the electroencephalogram can identify spectro-temporal phenotypes that reliably distinguish SWDs from convulsive seizures, indicating that (1) SWDs are normal, not to be falsely classified as nonconvulsive epileptic seizures; (2) the automated detection of convulsive seizures over months revealed rare post-traumatic epilepsy with low seizure frequency; (3) early and late (epileptic) seizures were indistinguishable within and across rats, thus suggesting similar underlying neuronal circuits and ictogenic pathways. This commonality, however, may also obscure important differences between seizure types; (4) convolutional neural network modeling may facilitate objective comparison of seizures within and between laboratories, supplementing subjective expert visual classification, and (5) the rarity of injury-induced epilepsy argues fluid percussion injury is poorly suited for effectively testing anti-epileptogenesis therapies.

## Significance Statement

CNN/EEG modeling can identify fundamental spectro-temporal phenotypes that reliably distinguish SWDs from convulsive seizures, indicating that SWDs are normal EEG, not to be falsely classified as nonconvulsive epileptic seizures. Accurate, automated detection of convulsive seizures over months revealed PTE was rare after FPI, with low seizure frequency. Early and late (epileptic) seizures were indistinguishable within and across rats, thus suggesting similar underlying mechanisms and neuronal circuits. This commonality, however, may also obscure important differences between seizure types. CNN modeling can facilitate objective comparison of seizures across laboratories, supplementing subjective expert visual classification. The rarity of FPI-induced PTE, and the low seizure frequency in FPI-induced PTE, argues that FPI is poorly suited for effectively testing anti-seizure and anti-epileptogenesis therapies.

## Introduction

Epilepsy is a chronic neurological condition defined by spontaneous recurrent seizures (SRSs). While seizures in both humans and animal models are often identified by convulsions, seizures can also be nonconvulsive. Therefore, beyond convulsive behavior, seizure detection (particularly automated detection) and classification must rely heavily on electrocortical activity recorded by electroencephalography (EEG).

Defining an unequivocal epileptic phenotype in the EEG presents formidable challenges. The question “What is a seizure?” remains a topic of long-standing and ongoing debate in both clinical and preclinical research (Dudek and Bertram, 2010; D’Ambrosio and Miller, 2010). Seizure identification often relies on experienced interpretation of EEG waveforms, but significant variability exists. This ambiguity has important implications for understanding seizure mechanisms and evaluating therapeutic interventions. A contemporary example is found in the rat model of post-traumatic epilepsy (PTE) induced by fluid percussion injury (FPI; D’Ambrosio et al., 2004, 2005; Kharatishvili et al., 2005, 2006; Pitkänen and McIntosh, 2006; Pitkänen et al., 2007, 2009). In this model, two visually distinct EEG patterns often arise: (1) nonconvulsive spike-wave discharges (SWDs) associated with behavioral immobility (Rodgers et al., 2015) and (2) discharges evolving in frequency and amplitude that are often correlated with convulsive behavior (Kharatishvili et al., 2006). These observations critically challenge the hypothesis that SWDs are nonconvulsive seizures, versus normal EEG patterns.

To address these concerns, objective and quantitative (vs subjective and descriptive) classification of EEG events is needed. Recent advances in artificial intelligence (AI) and machine learning may offer a promising path forward. Deep learning using convolutional neural networks (CNNs) can automatically extract relevant features from large datasets. GoogLeNet, a pretrained CNN designed for image classification (Szegedy et al., 2015; Bangar, 2024), can be adapted for EEG pattern recognition through transfer learning. EEG waveforms are first converted into time–frequency “scalograms” (Peng and Chu, 2004), enabling CNNs to classify EEG events as image-based features.

Here, we applied transfer learning with a CNN to compare FPI-induced convulsive seizures (“early” and “late”) recorded in long-term continuous EEG versus EEG patterns common to rats with and without PTE and thus probably normal oscillatory brain activity. We focused on three key types of EEG events: (1) “early-onset” or acute nonepileptic seizures that occurred ≤5 d after treatment and electrode implantation, (2) “late-onset” seizures that occurred >5 d after FPI treatment (and define PTE), and finally (3) SWDs, which can have either generalized (i.e., bilaterally synchronous) or focal onsets and have previously been described in a wide range of normal and injured rats. SWDs with generalized onset have often been considered a model of absence seizures/epilepsy (van Luijtelaar and Sitnikova, 2006; Depaulis et al., 2016), while focal-onset SWDs—or “epileptiform electrocorticography events” (EEEs)—observed after FPI have been considered nonconvulsive focal or “partial” seizures characteristic of PTE (D’Ambrosio et al., 2009). Because SWDs have been observed in many normal rat strains (Kelly, 2004; Pearce et al., 2014; Taylor et al., 2019), their significance for PTE remains uncertain if not dubious (Rodgers et al., 2015).

We aimed to determine whether, with the help of CNNs, we could (1) discriminate different seizure types, such as “early” versus “late” seizures, (2) distinguish phenotypic differences between these seizures and normal nonepileptic SWDs, and (3) determine whether the normal SWDs observed in rats without detected PTE seizures (i.e., non-PTE rats) differ from SWDs in rats with PTE (i.e., with late epileptic seizures). A final critical issue was whether nonconvulsive events could be objectively identified as either a seizure or as a normal brain oscillation, either by the CNN model or visual observation.

## Materials and Methods

### Experimental design and statistical analyses

#### Animals

The cohort of animals used in this study was drawn from pilot studies investigating the influence of preinjury stress on FPI-induced epileptogenesis. A total of 61, 2-month old, virus-free male Wistar rats (Harlan Laboratories) were used. Animals were housed in pairs under controlled temperature (23 ± 3°C) and lighting (12 h light/dark cycle), with *ad libitum* access to food and water. All procedures were performed in accordance with the American Physiological Society's Guiding Principles in the Care and Use of Vertebrate Animals in Research and Training and with University of Colorado Institutional Animal Care and Use Committee (IACUC) guidelines for the humane use of laboratory rats in biological research (protocol approved by IACUC).

#### Fluid percussion injury

**Figure 1.**
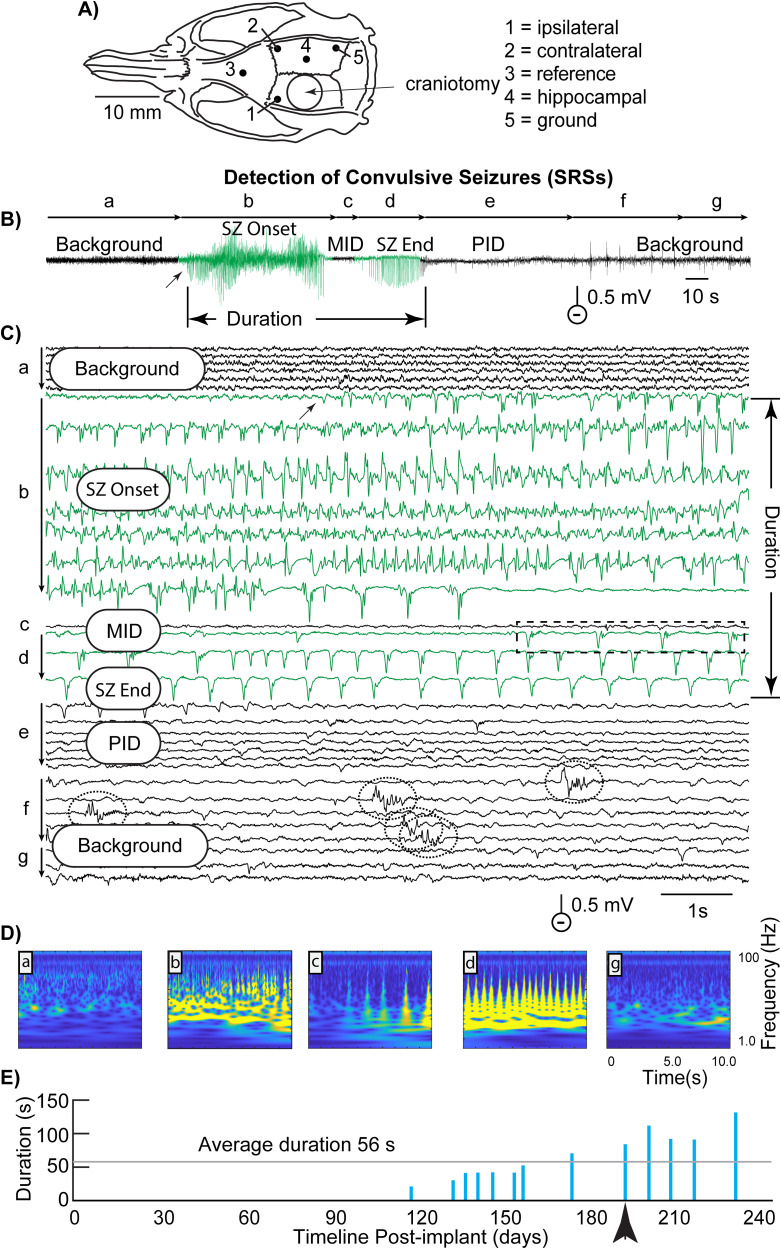
Detection of seizures (SRSs) with CNN. ***A***, Site of craniotomy with surrounding electrode locations. ***B***, ***C***, The typical electrical activity of a seizure is shown in time-compressed form in ***B***, and ***C*** illustrates the activity in expanded time scale. A seizure usually began with EEG spikes of variable frequency (***b***), often followed by mid-ictal depression (***c***; MID) and then resumption of lower frequency ictal spiking (***d***). After post-ictal depression (***e***; PID), background activity resumed and often showed inter-ictal epileptiform discharges (IEDs) or EEG “spikes” (dotted ovals; ***f***). ***D***, Scalograms of the different seizure phases used to train the CNN. Each scalogram is computed with a continuous wavelet transform (CWT) with time (0–10 s) and frequency (1–100 Hz) represented on the *x-* and *y*-axes, respectively. Scalograms are better than spectrograms for representing nonstationary signals with transient features (like EEG, seizures, and SWDs). ***E***, The post-implant timeline shows 13 recording intervals (duration, 0.5 h each) containing detections of seizure activity, which was only detected between 110 and 230 d (late seizures) in this rat. Vertical lines represent intervals where one or more seizures were detected. Line height reflects the total duration of seizure activity in seconds for that interval. Total seizure duration in seconds for each data interval was plotted on the *y*-axis. In this particular rat, a total of 13 EEG intervals with seizures occurred before the electrode cap was lost. The arrow under the timeline indicates the seizure illustrated in ***B*** and ***C***.

FPI-treated rats were anesthetized with isoflurane (3.5% induction, 2.0–2.5% maintenance), and a 5 mm craniotomy (Fig. 1*A*) was performed over the left hemisphere (AP: −4.0 mm, ML: 3.0 mm). A Luer-Lock hub was fixed to the skull and connected to a pneumatic fluid percussion device (PV820 Pneumatic PicoPump; World Precision Instruments). Once animals regained a forepaw withdrawal reflex, a mild fluid impact (30–34 psi, 20 ms) was delivered. Sham-controls underwent identical procedures, but without the impact. Righting time was ∼20 min, and no mortality occurred.

#### Preinjury stress protocol

To examine a possible interaction of stress and injury, 26 rats underwent a 1 h session of preinjury stress, during which 1 s footshocks (1 mA) were delivered every 5 s. FPI was administered 24 h later. Twenty-seven FPI rats were studied without prior stress and two sham groups (*n* = 9 stressed, *n* = 10 nonstressed) were also compared.

#### Chronic EEG and video monitoring

Rats were implanted with bilateral epidural electrodes (AP: 0 mm, ML: 4.5 mm), a reference screw (AP: 4 mm, ML: 1 mm), and a ground screw (AP: −6 mm, ML: 4.5 mm) 24 h after FPI surgery (Fig. 1*A*). A hippocampal electrode was also placed contralateral to the FPI-induced injury (AP: −4 mm, ML: 2 mm, DV: 2.0–2.5 mm). EEG was amplified (×500), digitized at 500 Hz, and recorded continuously for ∼182 ± 22 d, with synchronized video for behavioral scoring. EEG data were digitized and stored in contiguous 0.5 h intervals.

#### Visual seizure identification

Seizures were initially identified by visual inspection of EEG, supported by concurrent video. Convulsive seizures were identified by progressive EEG spiking (1–20 Hz), occasionally interrupted by mid-ictal depression (MID), and followed by post-ictal depression (PID) of the EEG. Seizure duration was measured from the onset of this activity pattern to the return to baseline; thus, it did not include PID as part of the seizure. Behavioral severity and latency since implant were recorded. Nonconvulsive SWDs were identified by their stereotyped oscillatory spike-wave morphology.

#### CNN-based pattern classification

For all analyses, CNNs trained on seizure or SWD examples from one subset of animals were applied to EEG data from different animals without subject-specific retraining, providing cross-animal validation of model performance.

CNN workflow: (1) visual identification and labeling of EEG events, (2) segmentation into 10 s epochs, (3) conversion to time–frequency scalograms using continuous wavelet transforms, (4) transfer learning with a pretrained CNN architecture (GoogLeNet), (5) training/validation on labeled examples, and (6) application to continuous EEG for automated detection with a fixed confidence threshold.

A CNN pretrained for image classification (GoogLeNet) was used with transfer learning to classify EEG events (Kotloski, 2023). Multiple 10 s segments of raw EEG data were visually selected for examples of convulsive seizures, SWDs, and background activity. These 10 s samples were transformed into scalograms—wavelet-based images representing time–frequency dynamics. Once trained, the CNN was tested across various rats and time points.

Two detection strategies were used for convulsive seizures: (1) CNN trained on all verified convulsive seizures across rats and then tested on individual rats in the group to evaluate cross-subject robustness. (2) CNN trained on either late-onset seizures (>5 d post-implant) or early-onset seizures (<5 d post-implant). The latter (“early” seizures) appeared to result from the electrode implantation surgery and/or the craniotomy in the FPI or sham-control surgery. The CNN was tested again for detections as a function of time after electrode implantation and FPI or sham treatment.

SWD classification involved similar procedures. To determine whether SWDs were different in FPI-PTE animals or the same in all rats, the CNN was trained on SWDs from rats with late seizures (i.e., PTE) and tested across all groups, including FPI-treated rats without late seizures and sham-control rats. We assumed that if the CNN could not differentiate between SWDs of control versus FPI-treated rats, particularly PTE rats, they are normal EEG oscillations and not PTE seizures.

### Statistical tests

Data were analyzed using ANOVA to assess the effects of injury, stress, and epilepsy status on seizure and SWD rates. Post hoc comparisons were performed using two-sample *t* tests. Statistical significance was set at *p* < 0.05, and data are presented as mean ± SEM.

### Software accessibility

All code for CNN modeling and seizure detection was written by the corresponding author, DSB, for use in the common MatLab programming environment. Analysis was performed in MATLAB (R2025a). These program files are available for download at GitHub https://github.com/dbarth-tech/ML-seizures.

### Data accessibility

These program files are provided along with exemplary EEG data files to evaluate software functionality. Each data file is provided with associated code used for reading it in the correct format.

## Results

### Seizures

#### Visual identification of seizures

Prior to use of the CNN model for implementation of automated seizure detection, all of the EEG recordings from every rat were visually examined for seizures (“manual identification”) with blinded procedures performed by naive experimenter (coded randomized filenames unrelated to experimental condition) by displaying all successive half-hour intervals (i.e., 0.5 h) of EEG (up to ∼7 months of data or ∼10,000 half-hour intervals each) from a single electrode as a raster plot composed of contiguous 10 s segments (Fig. 1*B*; 5 min of EEG shown here).

#### Seizure properties

A typical EEG-recorded seizure evolved through one or more distinct types or patterns of electrical activity, which is an important feature for visual identification and confirmation of a seizure (Fig. 1*B*,*C*). “Seizure onset” was characterized by large-amplitude spiking (approximately >2 times the amplitude of preceding background activity; Fig. 1*B*,*C*, “SZ Onset”). Repetitive spikes varied in amplitude and waveform, but the end of the seizure was determined by attenuation of spiking and a return of amplitude to that of the previous background activity. During the middle of a seizure, the EEG could suddenly undergo either a marked reduction in amplitude or a full interruption of the electrical activity for variable time periods (i.e., “mid-ictal depression”; Fig. 1*B*,*C*, “MID”). After an MID, electrical seizure activity could re-appear for a variable duration, usually lasting many seconds or tens of seconds; this activity comprised the latter part of the seizure (Fig. 1*B*,*C*, “SZ End”). A distinct phase of “post-ictal depression” (PID) could last for many more seconds (Fig. 1*B*,*C*) and was sometimes followed by inter-ictal “multi-spike bursts” (Fig. 1*B*,*C*, dotted ovals) before returning to background activity (Fig. 1*B*,*C*). While specific patterns of spike-frequency/pattern variation and the durations of MID (when present) and PID (usually present) varied across rats, the general patterns appeared quite similar. The seizures were also similar to other rat models of acquired epilepsy, such as from the CCI-TBI model (Statler et al., 2009), the postnatal day 7 hypoxia-ischemia model (Kadam et al., 2010), the maternal stress/terbutaline model (Bercum et al., 2015; Smith et al., 2020), and post-SE kainate and pilocarpine models (Williams et al., 2009; Smith et al., 2018), strongly suggesting a ubiquity that may be model independent.

#### Approach for training the CNN on seizures

To use a CNN pretrained for automated image classification (GoogLeNet), we first converted the 10 s segments of visually identified seizures (also SWDs, shown later) and background samples (Fig. 1*B*,*C*) into images, as time–frequency scalograms (Fig. 1*D*). Each scalogram was computed with a continuous wavelet transform (CWT; Peng and Chu, 2004), with time (0–10 s) and frequency (1–100 Hz) represented on the *x*- and *y*-axes, respectively. Scalograms are better than spectrograms for representing nonstationary signals with transient features (like EEG, seizures, and SWDs). In the example of Figure 1, ∼20–30 visually identified seizure scalograms versus background scalograms from a single rat were used to perform “transfer learning” with the CNN, with half the images randomly chosen for training and the other half used for validation.

#### Automated seizure detection

Following training, the 180 10 s segments from each 0.5 h EEG interval recorded for a given animal were converted to 10 s scalograms for experimental classification with the CNN. A duration of 10 s was chosen for classification of EEG segments because it presented an optimal trade-off between detection sensitivity, temporal resolution, and processing time. The 10 s analysis segments retained sensitivity to time periods of seizure activity that only lasted a fraction of 10 s (i.e., Fig. 1*Cd*, dashed box; Fig. 1*Dc*); that is, seizure activity was reliably detected even when it lasted for less than half of the 10 s segment. Total seizure duration (the cumulative time of automated ictal detections per 0.5 h recording interval) was logged in seconds (Fig. 1*E*).

#### Total duration of ictal activity per 0.5 h analysis interval estimated from CNN detections

In the highlighted seizure of Figure 1*B*,*C*, the associated vertical detection line (Fig. 1*E*, arrow) is placed at 200 d post-implant with a height of 100 s, reflecting the total duration of seizure activity detected within that particular 0.5 h analysis interval. As can be seen in Figure 1*C*, this represents seven data segments of 10 s duration and additional segments of ∼6 s at the beginning and end of seizure onset, and the beginning of MID, where seizure activity was identified by the CNN (shown in raster plot as 10 lines highlighted in green). The raster line during MID in Figure 1*B*,*C* is black because it was nearly flat and not detected by CNN as ictal. Detection timelines such as that shown in Figure 1*E* displayed the temporal distribution of seizure activity post-implant. By selecting a given marker (vertical line), a raster plot of each relevant seizure was provided for visual analysis of real versus false (noise) detections as well as more precise quantification of the durations of different seizure phases. In this example, the vertical line represents a total seizure duration of 88 s from “SZ Onset” to “SZ End.” To summarize the overall seizure activity detected in a given rat, we averaged across seizure times of all detections) per 0.5 h interval (in this rat, the average time was 56 ± 12.7 s).

#### Training the CNN on all seizures

**Figure 2.**
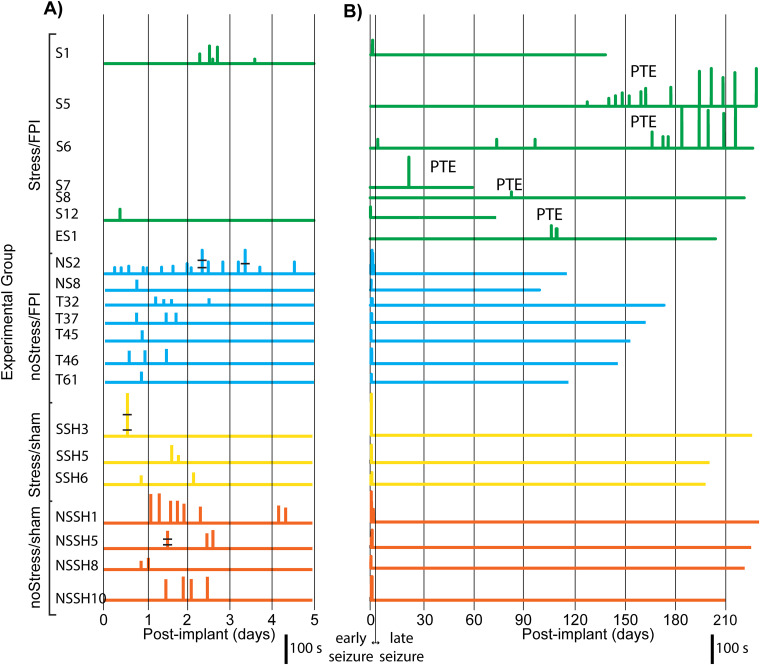
Seizure detection timelines with CNN trained on all (“early” and “late”) seizures. Detection timelines of rats are only shown for those rats with early or late seizures (21 out of 61 rats or 34%). ***A***, Before injury, FPI rats were exposed to either inescapable (S1–S12) or escapable shock (ES1; green traces), no-shock (NS2–NS6 and T32–T61; blue traces), stress with sham surgery (SSH3–SSH6; yellow traces), or no-stress with sham surgery (NSSH1–NSSH10; brown traces). Seizures were considered “early” if they occurred ≤5 d post-implant and “late” if they occurred after 5 d. Each vertical line is associated with a 0.5 h data interval in which seizure activity was detected. The line height reflects the total seconds of seizure detected in the segment. This was usually a single seizure but sometimes the sum of multiple seizures within the segment (i.e., Rat SSH3 had three seizures, demarked with black bars). Because the early seizures occurred ≤5 d post-implant and thus showed considerable overlap in the graph of post-implant latency (see above), the 5 d periods for each animal with “early” seizures are plotted with an expanded time scale for better visualization. ***B***, Similar to ***A*** but depicting the full post-implant timescale.

When the CNN model was trained using scalograms computed from all visually identified seizures (*n* = 99), the detection accuracy was ∼95% (94 seizures correctly detected). There were eight false detections during the same time period, when the detection confidence was set to >99% (a setting used throughout all analyses). Files reflecting a representative variety of background EEG were identified and included in the training. For cases where visual review indicated a false positive or a missed seizure detection, these traces were added to the noise- and seizure-sample files, respectively, and then, the CNN was retrained. Timelines of these detections for the separate rats are shown in Figure 2*A*,*B* (only timelines of rats with seizures are shown). As noted earlier, each seizure detection is plotted as a vertical line with the height reflecting the total duration of detected seizure activity during each 0.5 h data interval under examination. Most detection lines reflect single seizures of variable durations (including MID when present) at the given post-implant latency (Fig. 1*E*). On occasions where more than one seizure occurred during the 0.5 h data interval, the seizures are marked with black bars (Fig. 2*A*; rat SSH3 had a cluster of 3 seizures in one 0.5 h interval).

#### Early versus late seizures

Of the 99 convulsive seizures, over two-thirds (*n* = 68) occurred ≤5 d post-implant) and thus were considered “early,” while the rest of the seizures (*n* = 31) were observed >5 d post-implant and thus defined as “late” and indicative of PTE (Fig. 2*B*). Because the early seizures occurred ≤5 d post-implant, thus showing considerable overlap in the graph of post-implant latency, the 5 d periods for each animal with “early” seizures are separately plotted (Fig. 2*A*) with an expanded timescale for better visualization. We separated early from late seizures, because most of the animals with seizures had seizures that typically resolved within a few days (here, ≤5 d). Because these “early seizures” were often present in rats without FPI (i.e., they occurred in both FPI and sham-control non-FPI rats), they seemed to be associated with the electrode-implant surgery and craniotomy (however, 70% of the sham rats did not have any seizures). This contrasted to late seizures, which could persist for several months after the implantation surgery procedure and only in FPI-injured rats. Spontaneously occurring late seizures (i.e., SRSs) are generally considered the actual definition of PTE and that definition was also used in this study (Fig. 2*B*; see timelines for rats S5, S6, S7, S8, and ES1). Only two rats (S5 and S6) had several late seizures (the usual clinical definition of epilepsy is two or more unprovoked seizures). Two of the rats only had a single late seizure (S7 and S8), and one rat only had three late seizures (ES1); these three rats were also included in the PTE group. We included the two rats with only one SRS because we are assuming that other SRSs would have occurred if longer periods of continuous recording had been undertaken (similar to the experiments of Ndode-Ekane et al. (2024). Although Rat S7 was only monitored continuously for 2 months, both Rats S8 and ES1 were recorded continuously for >6 months; therefore, the potential limitation of a relatively short period (i.e., 2 months) of continuous recording only applied to one of the PTE rats. Thus, a total of 5 rats out of 61 (8.3%) developed PTE, and this proportion could be seen as a low estimate of the incidence of PTE, because we could have theoretically missed some late seizures, or SRSs could have occurred if we had recorded continuously for longer periods. None of the five rats that we have considered to have potentially developed PTE had any early seizures, including the two rats with many “late” SRS. All of the “no stress FPI-treated” rats had early seizures without any evidence of PTE, even though continuous recording was successful for at least 3–5 months. Importantly, all of the sham-control rats had early seizures, and most of these control rats also had seizures that persisted for more than a single day (Fig. 2*B*); therefore, although the sham-control rats had early seizures and were recorded continuously for 6–8 months, none of them showed any evidence of PTE checked with both CNN and follow-up visual examination (unblinded following analysis).

#### Preinjury stress

As can be seen from the timeline plots of Figure 2, only rats receiving both preinjury stress and FPI (S1–ES1) showed late seizures, typically commencing >60 d post-implant. While only rats with preinjury stress developed PTE, the effect of stress did not reach significance (*p* = 0.063).

#### Durations of visually measured convulsive seizures

**Figure 3.**
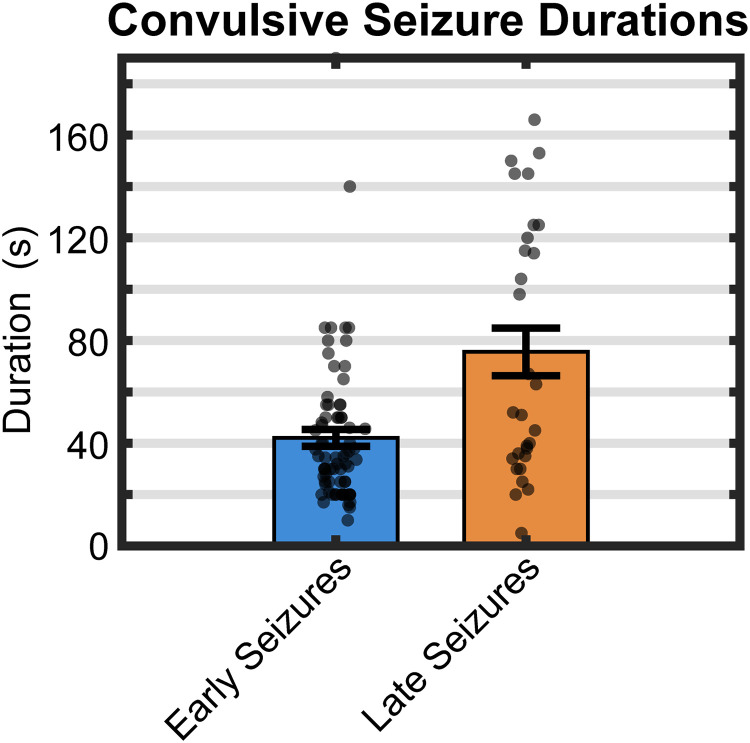
Distribution of visually measured early and late seizure durations. Seizure durations based on visual measurement of combined phases following CNN detection (SZ Onset, MID, SZ End) were shorter for early versus late seizures, averaging 52.04 ± 7.3 s and 83.1 ± 12.9 s, respectively (*t*_(46)_ = −2.49, *p* = 0.008).

Because a single seizure with MID could be mistaken for two shorter seizures, duration was determined through visual inspection of detected events. All of the 99 seizures visually identified across rats (Fig. 2) were considered convulsive (as verified by accompanying time-locked video). Only 26 of these seizures had MIDs. The MIDs varied from 10 to 180 s in duration (average 45.8 ± 9.6 s). The total duration of each seizure was typically measured (using visual analysis following detections, as noted above) from “SZ Onset” to the beginning of PID, which included the MID and “SZ End” periods, when present. Seizure durations determined in this way averaged 62.4 ± 6.6 s. Of the 73 seizures without MIDs, seizure durations were determined as those of “SZ Onsets” alone, which averaged 37.89 ± 2.6 s. Total seizure durations (“SZ Onset,” MID, “SZ End”) were shorter for early versus late seizures, averaging 52.04 ± 7.3 s and 83.1 ± 12.9 s, respectively (*t*_(46)_ = −2.49, *p* = 0.008; Fig. 3).

#### Training the CNN on early versus late seizures

**Figure 4.**
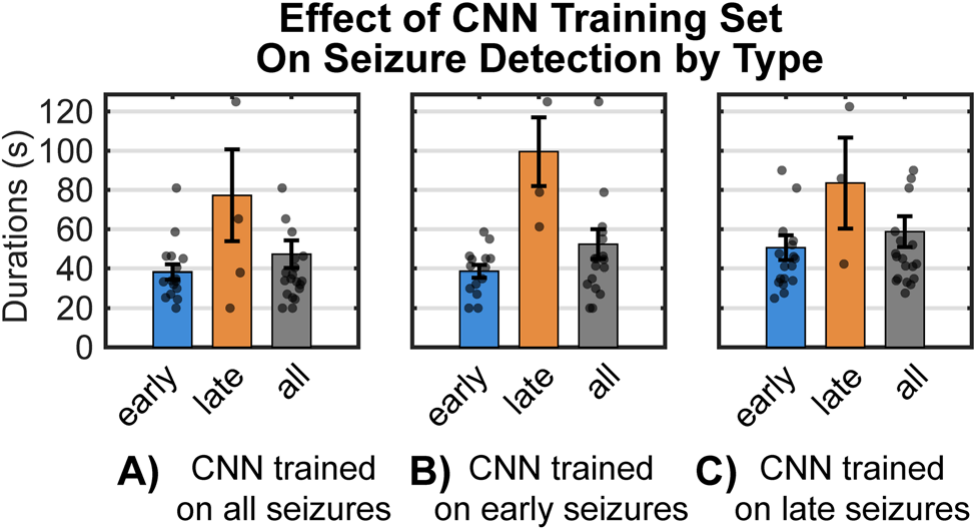
Durations of seizure detections with different CNN training. If early and late seizures had fundamentally different spectro-temporal patterns, one would expect that training the CNN on early seizures but testing it on detecting late seizures and vice versa (“cross-over training”) would produce significantly different results. ***A***, To explore this, we first trained the CNN on all seizures and then tested it separately for detecting early, late, and all (early + late) seizures. The “seizure detection durations” (the average seconds of detected seizure activity per 0.5 h recording interval) were shorter (42.08 ± 3.29 s) for early seizures and longer (75.58 ± 9.25 s) for late seizures (*t*_(35)_ = −3.4, *p* = 0.048) and (51.42 ± 3.78 s) when the CNN was tested on all seizures. This could be explained simply by the fact that early seizures were shorter than late seizures (Fig. 4) and thus presented fewer seconds of seizure time to be detected as a whole. However, when the CNN was trained only on early (***B***) or late (***C***) seizures, the relative detection times remained nearly the same. There was no main effect of post-implant seizure latency (early vs late; *F*_(2,106)_ = 1.32, *p* = 0.27) or training (*F*_(2,106)_ = 0.11, *p* = 0.89) and no interaction (*F*_(4,106)_ = 0.13, *p* = 0.97). These data indicate a generalization of the CNN model. Training the CNN on either seizure type produced a phenotypical seizure model that was equally applicable to all seizures. Regardless of differences in duration between early and late seizures, their spectro-temporal patterns are the same and the CNN preserves this similarity. These analyses directly test generalization by training on restricted seizure subsets and evaluating detection across animals and latency classes.

As noted above, training the CNN on both early and late seizures (i.e., all seizures) produced 95% detection accuracy, which could be expected given that detections included the same seizures the CNN was trained on. To obtain a better understanding of the generality of the model and the potential similarity of the seizures, we trained the CNN on “only early seizures” and then tested it on “all seizures” (Fig. 4*B*, “CNN trained on early seizures”). The average seizure durations (seconds of detected seizure activity per 0.5 h recording interval, averaged across all intervals) for both early and late seizure detections individually were nearly identical to those when the CNN was trained with all (i.e., both early and late) seizure types (Fig. 4*A*, “CNN trained on all seizures”). Similar results were obtained when the CNN was exclusively trained on only late seizures (Fig. 4*C*, “CNN trained on late seizures”). The durations of seizure detections for early seizures were significantly shorter (42.08 ± 3.29 s) than late seizures (75.58 ± 9.2 s; *t*_(35)_ = −3.4, *p* = 0.048) regardless of CNN training (two-way ANOVA indicated no main effect of post-implant latency or training and no interaction). This finding is potentially important for at least two reasons. First, it provides evidence that early and late seizures do not have any significant spectro-temporal (i.e., waveform pattern) differences, and training on either type of seizure (i.e., on early seizures vs late seizures) detected both seizure types equally well. But perhaps more important, this result suggests that training the CNN on different subsets of seizures results in a phenotypical model that can detect seizures in an expanded population that was not included in the training. Thus, it appears that the CNN may be attuned to a common convulsive seizure phenotype.

To explicitly test model generalizability and to avoid inflating performance by training on all available seizures, we trained CNNs on restricted seizure subsets (early-only or late-only) and evaluated detection across animals and across latency classes (Fig. 4). Training on either subset yielded detection durations comparable to CNNs trained on all seizures, indicating that the model captures conserved spectro-temporal seizure features rather than animal-specific or latency-specific patterns.

### Spike-wave discharges

#### SWD properties

**Figure 5.**
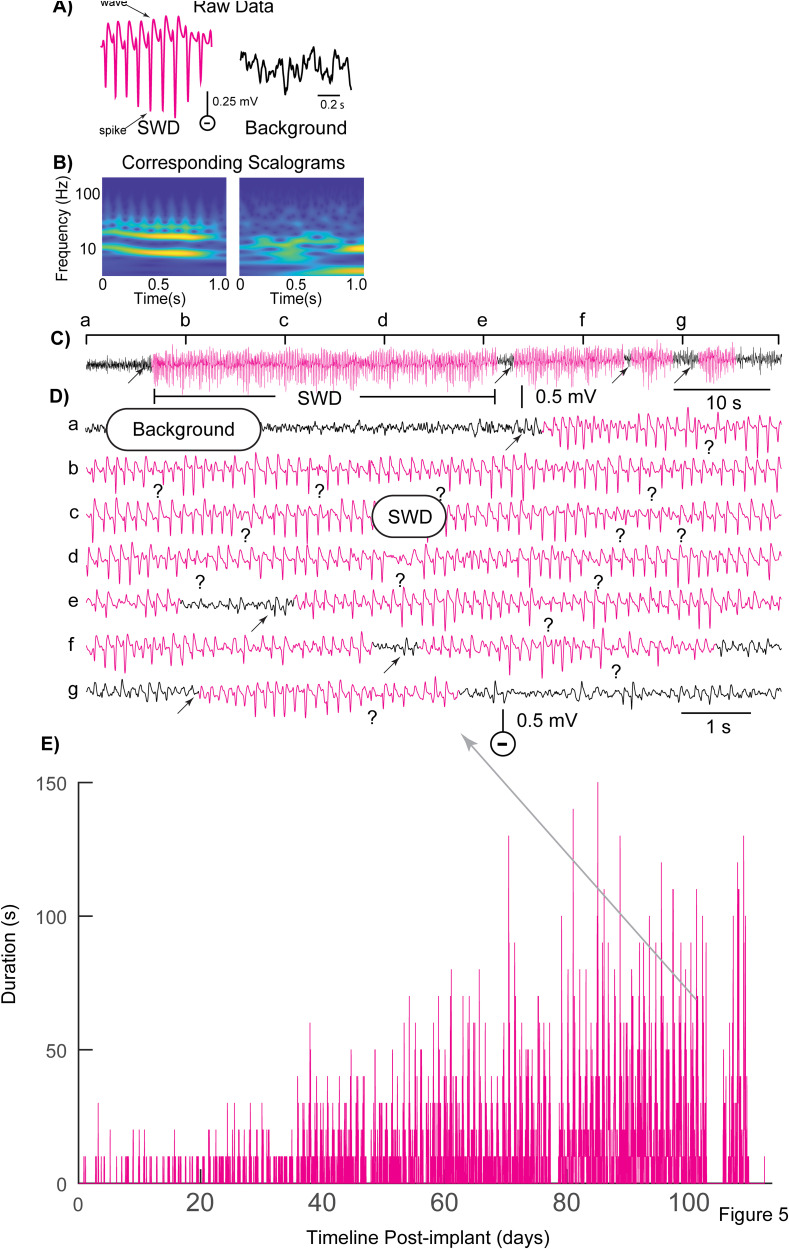
Detection of SWDs with CNN. ***A***, One-second sample of a typical SWD (9 Hz) compared with background EEG with corresponding scalograms (***B***) used to train the CNN. ***C***, Compressed time scale plot of EEG containing SWD detections (magenta). ***D***, Expanded time scale plot of data from ***C*** in raster format. SWD epochs could be as short as 3 s (***g***) but often continued for 10's of seconds (***b–d***). While we have attempted to indicate SWD onsets with arrows in the raster plot, this was not always straight forward due to a substantial waxing and waning in amplitude over the course of what otherwise would be a continuous SWD epoch. To emphasize this, we have marked possible/ambiguous onsets or breaks in continuous SWD with a question mark below the recording. ***E***, Post-implant timeline shows that SWD detections were apparent even in the earliest days post-implant but grew in duration with increasing post-implant latency. Unlike convulsive seizures, where the vertical bars usually reflected single seizure durations, the ambiguity of the SWD onsets led to the need to calibrate the height of each vertical bar to the total number of seconds of detected SWD activity per 0.5 h recording interval.

Figure 5, *D* and *E*, depicts an example of several consecutive SWD epochs occurring over a 70 s time period. Several features of SWDs made them clearly distinguishable from convulsive seizures. First, they were not associated with convulsive behaviors; instead, they were accompanied by a lack of movement and/or interruption of ongoing movement (Wiest and Nicolelis, 2003). Most apparent was a stable spectral frequency of 7–12 Hz (Sitnikova et al., 2009; Sitnikova, 2017; Van Luijtelaar, 2020) and an oscillatory “spike-wave” morphology (Fig. 5*A*). SWDs typically attained full amplitude suddenly (Fig. 5*C*,*D*, arrows) and often displayed an occasional and repetitive waxing and waning in amplitude throughout each period, before ending suddenly. In contrast to convulsive seizures, SWDs did not show signs of MID or PID associated with relatively prolonged amplitude depression; instead, the SWDs often increased in amplitude (“waxing”) and then briefly decreased back to baseline levels (“waning”) until the end of the overall SWD (Fig. 5*C*,*Da*,*e*,*f*,*g*), thus creating a scenario where it was difficult to differentiate one longer SWD from multiple shorter ones (Fig. 5*D*; ambiguous separations between SWD epochs labeled with “?” below the recording). For this reason, it was not possible to accurately compare durations of SWD epochs with those of seizures. However, it was possible to obtain an estimate of relative SWD durations by averaging the summed 10 s segments flagged by the CNN as SWDs within each 0.5 h data interval as a function of post-implant latency (Fig. 5*E*). Detected SWD duration as a function of time after implantation appeared to progressively increase from 10 s (i.e., a single 10 s detection) immediately after implant to ∼100 s 90 d later (∼10 10 s detections). Finally, as previously noted in other reports (Rodgers et al., 2015), SWDs were equally common in both sham-control (i.e., not FPI-injured) and experimental FPI-treated rats.

#### CNN discrimination of SWDs from convulsive seizures

**Figure 6.**
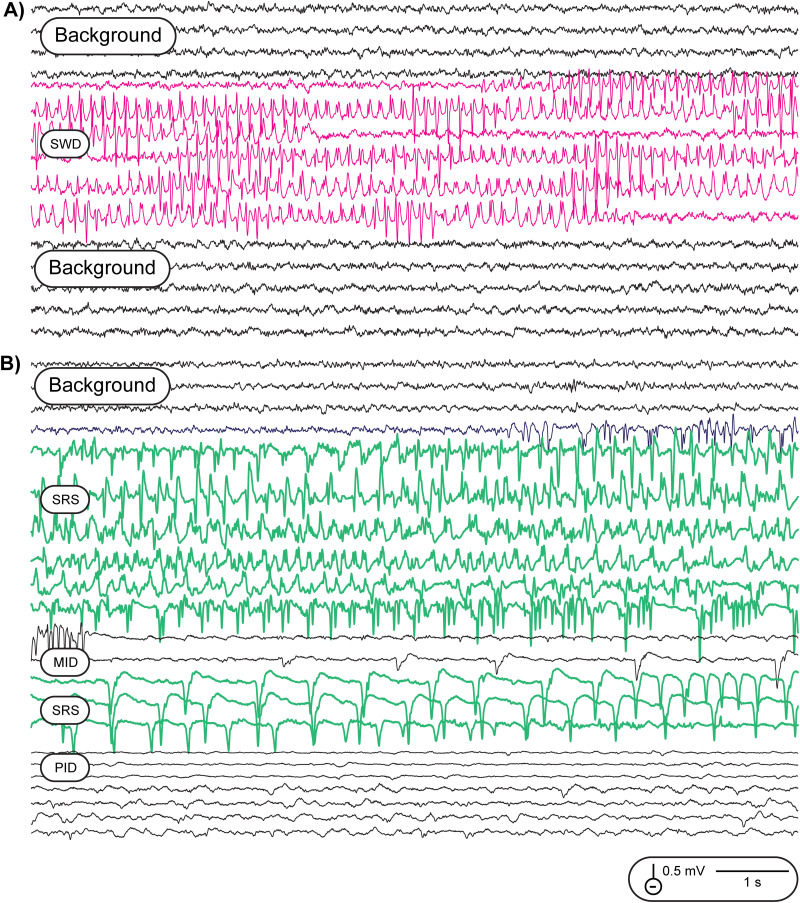
Discrimination of SWDs from convulsive seizures with CNN. Training the CNN on SWDs versus convulsive seizures, in the same rat or across rats, yielded two distinct model phenotypes. ***A***, The CNN model trained to detect SWDs in the rat shown in Figure 5 was applied to SWD detection in a different rat shown here. SWD detection was clearly generalizable to this rat and distinguished SWDs from background EEG. The frequency and waveforms in each example were nearly identical. ***B***, We then took the CNN model trained for convulsive seizure detection in the example shown in Figure 1 and applied it to the same rat shown here for SWD detection. As in all rats with convulsive seizures, they also displayed SWDs. Even though the CNN was trained on seizures from another animal, the CNN was equally sensitive to detecting all phases of the SRS here, and there was no overlap of SWD versus SRS detections.

Training the CNN on SWDs versus convulsive seizures, in the same rat or across rats, yielded two distinct model phenotypes. The CNN model trained to detect SWDs in the rat shown in Figure 5 was applied to SWD detection in a different rat shown in Figure 6*A*. SWD detection was apparently generalizable to this rat and distinguished SWDs from background EEG. The frequency and waveforms in each example were nearly identical. We then applied the CNN model trained for convulsive seizure detection in the example shown in Figure 1 and applied it to the same rat shown here for SWD detection. As in all rats with convulsive seizures, they also displayed SWDs. Even though the CNN was trained on seizures from another animal, it appeared equally sensitive to detecting all components of the SRSs here, and there was no overlap of SWD versus SRS detections.

#### Are SWDs normal?

These points argue that SWDs are not nonconvulsive seizures, but that does not mean they are necessarily “normal” (i.e., nonepileptic) brain oscillations. Although SWDs appeared distinct from convulsive seizures, some features could still be directly altered by changes in cortical circuitry in and around the site of FPI injury. This raises the question: Do SWDs from sham-control rats have different phenotypes from FPI-injured rats, and rats with FPI-induced PTE? If so, while perhaps not representing actual seizures, SWDs may not always be “normal brain oscillations”; instead, these SWDs could be altered by the FPI and or PTE and thus different from normal SWDs. If the SWDs were altered during PTE, they could hypothetically serve as a biomarker for the development of PTE.

Here, the term “normal” is used to denote nonictal activity that does not predict or define post-traumatic epilepsy, rather than implying an unperturbed or injury-free brain state. Although electrode implantation and craniotomy can influence cortical network dynamics, the comparable SWD detections across sham-control and FPI-treated animals argue against interpreting SWDs as epileptic seizures or biomarkers of PTE in this context. If SWDs arose from procedures such as electrode implantation in both sham-controls and FPI-treated rats, one might expect that FPI would affect the SWD waveforms, but the FPI had no detectable effect on SWDs. Because electrodes are required to detect the SWDs, it is difficult to show that SWDs do not originate from the recording procedure itself, but this concern would apply to all other studies on SWDs, including those claiming that SWDs represent absence seizures.

#### Total SWD detection durations across treatment groups

**Figure 7.**
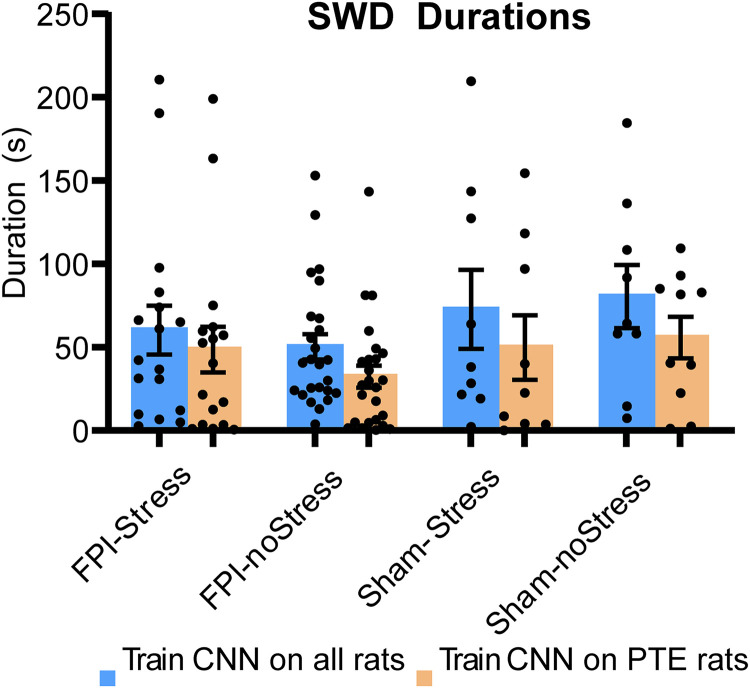
Average SWD durations measured across experimental groups as a function of type of CNN training. SWDs were abundant in rats with or without convulsive seizures. Observations that they are also abundant in control (noninjured) animals strongly suggests that they are not seizures and do not reflect epilepsy. However, while not being actual seizures, SWDs could indirectly reflect epileptic processes. The spectro-temporal properties of SWDs could be directly and systematically altered by changes in cortical excitability associated with epileptogenesis. If so, one might expect an epileptic SWD phenotype that could be reliably discriminated from “normal” SWDs and relied on as a biomarker for developing epilepsy. To test this possibility, we divided our rats into four groups: FPI-Stress, FPI-noStress, Sham-Stress, and Sham-noStress. The total SWD durations were computed for each of the four groups with the CNN trained on SWDs from all rats (blue) versus just rats with PTE (orange). The total SWD durations (i.e., detection times) in PTE rats (54.26 ± 8.37 s) appeared diminished compared with uninjured sham animals (77.22 ± 17.71 s), but this difference did not reach significance (*t*_(28)_ = −1.45, *p* = 0.078). Additionally, preinjury stress produced no significant differences in SWD detection times in stressed (64.53 ± 12.65 s) versus unstressed (59.1 ± 9.99 s) animals (*t*_(42)_ = 0.375, *p* = 0.35). Two-way ANOVA also indicated that there were no significant main effects of stress (*F*_(1,39)_ = 0.2, *p* = 0.65) or PTE (*F*_(1,39)_ = 2.85, *p* = 0.10) on SWD detections (i.e., total SWD duration). These results indicate that SWDs are normal brain oscillations and do not reflect epilepsy.

As with convulsive seizure detection, we examined this possibility by training the CNN with SWD samples from all rats, regardless of experimental condition, to see if total SWD durations (durations of detected SWDs averaged across rats within a treatment condition) differed across treatment condition. As can be seen in Figure 7 (blue bars), total SWD durations appeared largely unaffected by either stress or FPI. The total SWD durations (i.e., detection times) in FPI rats (54.26 ± 8.37 s) appeared diminished compared with uninjured sham animals (77.22 ± 17.71 s), but this difference did not reach significance (Fig. 7; *t*_(28)_ = −1.45, *p* = 0.078). Additionally, preinjury stress produced no significant differences in SWD detection times in stressed (64.53 ± 12.65 s) versus unstressed (59.1 ± 9.99 s) animals (*t*_(42)_ = 0.375, *p* = 0.35). These results quantitatively support the observation that SWDs are not a manifestation of PTE or altered by FPI-induced injury or preinjury stress, since they appeared identical in both experimental and control rats.

#### Total SWD durations in rats with PTE

Since the average SWD durations were the same in both FPI and sham animals, it seems highly unlikely that they were seizures. However, as noted earlier, the possibility still remained that there could be different types of SWDs, such as epileptic versus nonepileptic SWDs. Rats with PTE (as determined by late convulsive SRSs) could present SWDs that are spectrally and temporally distinct from those of nonepileptic rats. Did SWDs in rats exhibiting late seizures in the FPI-Stress group (the only group to develop PTE) differ from the FPI-treated rats that did not have any detected late SRS (i.e., rats that apparently were nonepileptic rats but might have developed PTE if they had been monitored longer). If so, one would expect that training the CNN on the SWDs recorded only from PTE rats would result in significant differences in detection durations across groups when compared with the CNN model trained on all SWDs (independent of PTE). However, even when the CNN was trained exclusively on SWDs from PTE rats, differences in SWD detection durations (Fig. 7, orange bars) mirrored those when the CNN was trained on SWDs from all groups (Fig. 7, blue bars). This was true despite the fact that there was only a small number (*n* = 5) of PTE rats to sample for CNN training, and three of the five PTE rats only had one or three late seizures. Yet, there is always the possibility that, since PTE (defined by spontaneous late seizures) often did not emerge until months after injury, PTE could have been missed in a subset of rats, biasing the results. However, because the SWDs in the different groups of the present data could not be distinguished by machine learning with the CNN model, SWDs may be the same in experimental FPI-injured and sham-control animals. Therefore, we conclude that SWDs are not seizures and, instead, are normal EEG oscillations.

## Discussion

### Scope and generalization

The CNNs were trained and evaluated exclusively on tethered, high-bandwidth epidural EEG acquired with the present amplification and acquisition chain. We have not tested, and therefore do not assume, that model performance generalizes to miniature wireless/telemetry recording systems, where differences in bandwidth, quantization, reference configuration, and motion-related artifacts can substantially alter time–frequency structure and classifier behavior. Any deployment to wireless EEG would require prospective validation and, if necessary, retraining or domain adaptation using data acquired with the target system.

### Spontaneous seizures and SWDs in the FPI model of PTE

Visual analysis and CNN modeling provided two complimentary methods for detection and confirmation of early and late (i.e., epileptic) seizures. We found the following: (1) FPI may lead to PTE, but with a low incidence (8%) and (2) the ability to train on early seizures to detect late seizures, and vice versa, strongly suggests that these two types of seizures have similar if not identical phenotypes. A similar approach for SWDs, the CNN model provided a novel, objective, and quantitative quantification of total SWD duration over time and also showed that SWDs were virtually identical in both sham and FPI rats (with and without PTE). The robust presence of SWDs in all of the groups, independent of treatment, suggests that SWDs are not seizures and that SWDs are neither induced (or even altered) by FPI treatment nor a manifestation of PTE.

### SWDs are common, nonictal oscillations that may often be misclassified as nonconvulsive seizures

SWDs are consistently observed in virtually all laboratory rat strains studied (Coenen et al., 1992; Kelly, 2004; Pearce et al., 2014; Rodgers et al., 2015; Ndode-Ekane et al., 2024) and should be considered nonepileptic oscillations rather than seizures. Our comparisons across experimental conditions and CNN training regimes revealed no qualitative or quantitative differences between SWDs recorded from FPI-treated, PTE rats and those from sham-controls. This aligns with prior work documenting the ubiquity of SWDs in commonly used rat strains (Coenen et al., 1992; Kelly, 2004; Pearce et al., 2014; Rodgers et al., 2015; Ndode-Ekane et al., 2024). Several previous studies have analyzed behavioral correlates of these SWDs in normal rats (Wiest and Nicolelis, 2003; Taylor et al., 2017), independent of any brain injury or potential model of epilepsy. Because these similar SWD-associated behaviors occur in naive rats without implanted electrodes, it seems unlikely that electrode implantation procedures caused the SWDs. Although specific SWD subtypes, such as SWDs with superimposed fast ripples (SWDFRs), have been proposed as potential predictors of epileptogenesis (Kumar et al., 2021), we did not observe increased SWD rates or fast ripples in PTE animals. However, with our sampling rate of 500 Hz, fast ripples (typically 200–500 Hz; Kumar et al., 2021) may have been undersampled in the upper frequency limit. Moreover, CNNs trained to differentiate possible SWD subtypes failed to reveal separable phenotypes between injured and control groups. These interpretations differ from Kotloski (2023) who treated SWDs postinjury as equivalent to PTE seizures; the absence of uninjured controls in that study may have contributed to misclassification. Taken together, our findings (and the prior literature) support the view that SWDs are nonictal and should not be conflated with seizures or considered early biomarkers of epileptogenesis (Pearce et al., 2014; Rodgers et al., 2015).

### Automated seizure detection is feasible, and CNNs have advantages over other methods for extended monitoring

Establishing PTE requires sustained EEG surveillance. In this context, CNNs offer distinct advantages over prespecified feature classification: they reduce false-positive burden over long recordings, are less sensitive to gradual changes in signal statistics, and typically require fewer manual recalibrations once trained (Zhang et al., 2024; Wu et al., 2025). Even though the incidence of late convulsive seizures in our cohort was low, detections were robust and consistent with the known frequency of PTE in FPI models. Practically, this enables continuous, scalable analysis of months-long datasets—an essential capability for acquired-epilepsy studies.

### Conserved spectro-temporal features across early and late seizures—opportunity and caveat

A central observation is that seizures occurring early (post-procedural) and weeks-to-months later (defining as PTE) share similar if not stereotyped spectro-temporal signatures. CNNs trained exclusively on early seizures detected late seizures with high fidelity, and vice versa, demonstrating near-complete portability across latency and causal context. This suggests that both types of convulsive seizures in this model express conserved electrical dynamics and probably similar circuit activity, which the CNN captures as consistent and invariant features. One translational implication is that if CNNs trained in one paradigm apply to others, different injuries or etiologies may suggest shared electrophysiological properties.

At the same time, commonality can obscure fundamental mechanistic and clinically meaningful differences. To probe potential subtype-specific features—particularly between convulsive and nonconvulsive seizures—Activation Maximization (feature visualization) may generate synthetic scalograms that maximally activate each target class, alongside saliency-based methods (e.g., Grad-CAM) on real data. This combined approach can expose frequency-band emphasis, rhythmicity, and onset morphology that differentially drive classifier decisions, helping to distinguish conserved elements from subtype-specific signatures (Nguyen et al., 2019). We note preliminary evidence that models trained on FPI-associated seizures can detect seizures in a developmental epilepsy model (maternal stress + terbutaline; Bercum et al., 2015). With transfer learning, modest amounts of labeled data may adapt a pretrained CNN across models, which would aid studies where seizure onset is delayed and/or seizure frequency is low and random, thus making manual screening impractical (particularly with large multicenter datasets).

### CNN modeling improves objective and reproducible comparisons within and between laboratories

Deep learning provides a quantitative alternative to purely descriptive visual classification, supporting reproducible seizure identification across animals, cohorts, and laboratories. Using transfer learning, we achieved high accuracy for convulsive seizures spanning early-onset events related to surgical procedures and late-onset PTE-defining events. The portability across animals indicates that core spectro-temporal features are consistent despite variability in post-implant timing or other experimental conditions. This complements work in clinical and preclinical domains, thus demonstrating improved accuracy and reproducibility of deep learning-based EEG analysis (Roy et al., 2019). While CNNs do not eliminate human bias at the point of label creation, subsequent detections are governed by a fixed, quantitative decision rule rather than evolving descriptive templates, enabling empirical cross-study comparisons of seizure phenotypes. We add that the CNN model would have to be tailored to the recording system, since the current system has been ineffective with wireless recording systems, but this issue should be addressable with recording system-specific retraining on the CNN.

CNN modeling has reinforced the potential impact of data from FPI-induced brain injury versus sham-control animals. The prolonged recording periods required to assess the presence of bona fide PTE is challenging for human visual analysis. The concept of optimized training would allow potential lifetime studies, while optimizing specificity and sensitivity of seizure detection. This will be essential for translational research where rigorous well-powered preclinical trials can be done to test specific hypotheses about new anti-epileptogenesis therapeutic approaches. Our observation of a low percentage of FPI-treated rats could be related to the fact that even with 6–8 months of continuous recording, we would still only be recording less than half of the duration of a life of a typical laboratory rat, so a greater proportion of the rats could have developed epilepsy with additional time. Optimization and wide use of CNN models for seizure detection and classification would benefit further studies that could evaluate the development of seizures throughout the life span, thus allowing an analysis of factors such as age-at-injury on epileptogenesis.

### FPI yields relatively low rates of PTE, limiting utility for rapid anti-epileptogenesis screening

Our combined visual and CNN-based analyses indicate that lateral FPI can produce low rates of PTE, consistent with prior reports of low PTE incidence in mild-to-moderate TBI models and clinical cohorts (Annegers et al., 1998; Bolkvadze and Pitkänen, 2012; Ndode-Ekane et al., 2024). In contrast, PTE is more common after severe “open head” or penetrating TBI (Salazar et al., 1985; Kendirli et al., 2014). Given these rates, FPI may be poorly suited for practical screening of anti-epileptogenesis therapies, although it could be an important model for studying risk factors, delayed seizure emergence, and mechanisms of acquired epilepsy. Separately, our quantitative treatment of SWDs underscores that they are not induced or altered by FPI and should not be used as a surrogate endpoint for PTE.

### Limitations and future directions

We propose new directions to optimize efficacy, specificity, and sensitivity. Class imbalance and labeling: Low PTE incidence yielded few late seizures. Future work should include recording system and subject-specific fine-tuning to improve sensitivity. Interpretability and electrophysiology: We will extend Activation Maximization and saliency methods with frequency-band masking and counterfactuals to test causal contributions of specific time–frequency components to classification decisions (Nguyen et al., 2019). This may help differentiate conserved convulsive features from subtle markers of nonconvulsive seizures. Generalization across models and labs: Prospective cross-laboratory benchmarking will be necessary to quantify reproducibility and portability. Transfer learning should be explored to adapt pretrained models from one injury paradigm (e.g., FPI) to others (developmental, inflammatory, or chemo-convulsant), minimizing labeling burden (Bercum et al., 2015). Clinical translation: low-maintenance monitoring with CNN detection could support chronic EEG surveillance in clinical settings. Integrating motion/EMG artifact detectors may further reduce false alarms during ambulatory EEG (Roy et al., 2019; Zhang et al., 2024; Wu et al., 2025).

### Conclusions

Our findings indicate that (1) SWDs are common, nonictal oscillations and should not be conflated with seizures or biomarkers of PTE; (2) CNNs enable accurate, scalable detection of late convulsive seizures over months-long recordings and could outperform other approaches in extended monitoring; (3) early and late seizures share conserved spectro-temporal features captured by CNNs, with ongoing interpretability work aimed at revealing subtype-specific differences; (4) deep learning should facilitate objective, quantitative and reproducible seizure phenotyping across animals and laboratories; and (5) FPI produces relatively low incidence of PTE, constraining its use for rapid anti-epileptogenesis screening but potentially remaining valuable for mechanistic studies. Together, these results position CNN-based analysis as both a practical tool for long-term detection and a probe of the electrophysiological mechanisms of seizures.

## Synthesis

Reviewing Editor: Mark Sheffield, University of Chicago Division of the Biological Sciences Decisions are customarily a result of the Reviewing Editor and the peer reviewers coming together and discussing their recommendations until a consensus is reached. When revisions are invited, a fact-based synthesis statement explaining their decision and outlining what is needed to prepare a revision will be listed below. The following reviewer(s) agreed to reveal their identity: Kevin Staley. Note: If this manuscript was transferred from JNeurosci and a decision was made to accept the manuscript without peer review, a synthesis may not be available.

Overall, the reviewers view the manuscript as addressing an important and timely problem in epilepsy research: the objective differentiation of epileptic seizures from normal thalamocortical oscillations in long-term EEG recordings. Given the ongoing controversy, particularly in models of brain injury, about whether certain spike-wave discharges represent epilepsy or normal rhythmic activity, the demonstration that a convolutional neural network (CNN) can reliably distinguish these patterns is seen as both meaningful and potentially field-advancing. Robust, automated seizure detection tools are especially valuable for large-scale and longitudinal studies, and the central result, that machine learning can separate epileptic seizures from non-epileptic oscillations, is regarded as compelling.

At the same time, several concerns need addressing.

A primary issue relates to validation and generalizability of the CNN model. While high seizure detection rates are reported when all seizures were included in the training dataset, it is unclear how performance changes when only a subset of seizures is used for training. Demonstrating detection rates under partial-training conditions is important to establish that performance is not inflated by including all available seizure examples. More critically, it is not clearly shown whether the CNN was trained on one set of animals and then tested on an entirely separate cohort without any subject-specific data included in the training set. Such cross-animal validation is essential to demonstrate generalizability and to ensure that the model is not relying on idiosyncratic features of individual animals. Although there is a suggestion that this may have been done in a limited case, systematic quantification and clearer presentation of these results are needed.

A second conceptual concern involves interpretation of spike-wave discharges (SWDs). It is argued that SWDs represent normal EEG oscillations because they appear in both injured and sham-control animals. However, is it possible that electrode implantation and craniotomy themselves induce pathological activity? The observation that some non-injured animals exhibited early seizures attributed to surgical injury raises the possibility that SWDs in sham animals may also reflect procedure-related effects. Moreover, the progressive increase in SWD duration following implantation suggests a potential link between surgical injury and oscillatory changes. These issues warrant more cautious interpretation and fuller discussion to avoid circular reasoning in defining "normal" versus pathological activity.

In addition to these conceptual points, there are issues of clarity and presentation. Key methodological details are either insufficiently described or difficult to extract from the current text. In particular, the EEG recording technology, data acquisition pipeline, and data transmission process require fuller explanation. While surgical procedures and sampling parameters are described, readers need a clearer account of the recording system, signal processing steps, and how raw data were prepared for computational analysis. Similarly, the machine learning workflow is presented too superficially. The manuscript would benefit from a more transparent and stepwise explanation of model architecture, preprocessing, training strategy, validation procedures, and performance metrics. As written, the description risks giving the impression that a pre-existing image-analysis CNN was simply "fed" EEG data without sufficient adaptation or optimization, which likely understates the methodological rigor involved.

Reviewers also recommend clearer organization of experimental groups, terminology, and analytical parameters. Definitions of key terms and metrics should be consolidated, potentially in tables or clearly delineated sections, to help readers understand how seizures and oscillations were classified and why specific features were selected. Including discussion of alternative or complementary analytical metrics (e.g., rhythmicity measures) could further contextualize the approach, particularly given that visually, normal oscillations appear more rhythmically regular while seizures appear noisier and more disorganized. Clarifying how the CNN captures such distinctions would strengthen interpretability.

## Author Response

Mark Sheffield Reviewing Editor eNeuro Dear Dr. Sheffield:

We thank the reviewers and editors for their constructive evaluation of our manuscript entitled, Deep learning discriminates seizures from normal brain oscillations in the electroencephalogram of a rat model of post traumatic epilepsy. We have revised the manuscript to address the concerns raised in the editor's synthesis statement. All changes in the revised manuscript are indicated by highlighted text, and a clean copy has been provided.

Our response to the editor's synthesis of the reviewers' statements is shown below.

Sincerely, Daniel S. Barth (on behalf of all authors) Response to the Reviewing Editor and Reviewers 1. Validation, partial training, and generalizability Concern:

CNN performance may be inflated by training on all seizures, and generalization across animals and partial training conditions was unclear.

Response:

We have further clarified and expanded the section on the analyses that directly tested generalizability. As was shown in Figure 4 (Durations of seizure detections with different CNN training), CNNs were trained on subsets of seizures (early only or late only) and evaluated across animals and across latency classes. Detection performance was comparable to CNNs trained on all seizures, indicating that the model captures conserved spectro temporal seizure features (E1).

Cross animal validation is now stated explicitly: CNNs trained on one subset of animals were applied to EEG from different animals without subject specific retraining (E2). Figure 4 is now explicitly framed as a generalization test rather than an exploratory analysis (E1, E5).

2. Cross animal testing Concern:

It was unclear whether training and testing were performed on separate animals or cohorts.

Response:

The manuscript now explicitly states that CNNs trained on seizures or SWDs from one subset of animals were applied to EEG from different animals, providing cross animal validation without subject specific retraining (E2). This is described in the Methods and referenced in the Results (see Fig. 4).

3. Interpretation of spike wave discharges (SWDs) Concern:

SWDs were described as "normal," but surgical procedures could influence cortical activity.

Response:

We have clarified terminology to define "normal" as non ictal, non epileptic activity in the context of post traumatic epilepsy, rather than implying an unperturbed brain state (E4). We also acknowledge that electrode implantation and craniotomy may influence network dynamics. However, comparable SWD detections across sham control and FPI treated animals, and the inability of CNNs to distinguish SWDs by injury or epilepsy status, argue against interpreting SWDs as seizures or biomarkers of PTE (E4).

4. Methods clarity and CNN workflow Concern:

EEG acquisition and machine learning methods were difficult to extract, and the CNN workflow appeared superficial.

Response:

The Methods section has been revised to provide a concise, stepwise description of the CNN pipeline, including segmentation, scalogram generation, transfer learning, training/validation, and application to continuous EEG (E3). These revisions clarify that the CNN was adapted and validated for EEG specific classification rather than applied off the shelf.

5. Organization and interpretability Concern:

Clearer organization, definitions, and contextualization of CNN decision making were recommended.

Response:

Definitions and terminology have been consolidated, and figure legends have been revised to explicitly frame key analyses as tests of generalization across animals and seizure subsets (E5). The Discussion now more clearly distinguishes conserved seizure features from interpretability questions that remain future work.

Summary The revised manuscript now explicitly demonstrates CNN generalizability (E1, E2, E5), adopts a more precise and cautious interpretation of SWDs (E4), and provides a clearer description of EEG acquisition and machine learning methods (E3). We believe these revisions directly address the concerns raised in the editor's synthesis statement.
